# Social work practice education and training during the pandemic: Disruptions and discoveries

**DOI:** 10.1177/0020872820959706

**Published:** 2020-11

**Authors:** Toula Kourgiantakis, Eunjung Lee

**Affiliations:** University of Toronto, Canada; University of Toronto, Canada

**Keywords:** Blended learning, COVID-19 pandemic, field education, holistic competence, online learning, social work education, social work practice, virtual learning

## Abstract

The COVID-19 pandemic evoked a disruption to social work (SW) practice education and this brief note describes discoveries made in teaching SW practice virtually. One example is Virtual Practice Fridays, adapted to build SW practice competencies online, and another example is a re-designed course on cross-cultural SW practice using simulation-based learning.

## Introduction

Recently, the COVID-19 pandemic has evoked unprecedented and challenging circumstances for social work (SW) education and practice. We are faculty members in a graduate school of SW with extensive practice experience and pedagogical research on the development of practice competencies using various innovative methods of teaching SW practice. In this brief note, we will discuss how SW practice training has been disrupted by the pandemic in both the field and the classroom. This disruption evoked discoveries related to teaching SW practice virtually, and we will provide two examples of how we leveraged technology to teach SW practice.

## Disruption: Reduced SW practice training in the field and classroom

Developing SW practice competencies is complex and requires a combination of procedural competencies such as knowledge and skills, along with metacompetencies such as self-awareness and self-reflection (Kourgiantakis et al., 2020). Students also need to develop cross-cultural competencies, learn to link theory with practice, and apply social justice principles when working with clients (Lee et al., 2020). The American Educational Policy and Accreditation Standards (EPAS) describe a ‘holistic view of competence’ and emphasizes the importance of a competency-based approach where students ‘demonstrate the integration and application of the competencies in practice’ (Council on Social Work Education [CSWE], 2015: 6). Field education is the signature pedagogy for SW, aiming to ‘integrate the theoretical and conceptual contribution of the classroom with the practical world of the practice setting’ (p. 12). Even before the pandemic, field education was in ‘crisis’ due to budget cuts, limited resources in practice settings, increased enrollments, and fewer practicum opportunities (Ayala et al., 2018). Schools of SW have been trying to reduce the burden on field by providing practice opportunities in the classroom. To prevent the spread of the coronavirus, physical distancing measures were implemented in many countries, disrupting SW placements as well as in-person classroom training (Canadian Association for Social Work Education [CASWE], 2020; CSWE, 2020). Some practice settings were unable to continue supervising students, while others offered remote learning plans (CASWE, 2020). Like other schools of SW, our faculty had to unexpectedly complete their 2020 winter courses online and re-design subsequent courses for online format.

Amid these disruptions to student learning about SW practice worldwide, there have been other realities for students, faculty, and community members related to health, mental health, economic and employment security, caregiving, grief, loss, discrimination, oppression, and racism. Most of these realities existed before the pandemic; however, many have been amplified, and this period has once again highlighted systemic racism (Serhan and McLaughlin, 2020) and inequities in the social determinants of health (Lai et al., 2020; World Health Organization [WHO], 2020). In a survey conducted by the American CSWE (2020) on student perceptions of the impact of COVID-19 on their educational experience (*N* = 3564), 81 percent indicated that the pandemic has negatively impacted their mental health, and 65 percent indicated that it has affected financial security.

## Discovery: Online education and training for SW practice

As we grapple with these challenges and disruptions to teaching and learning, SW programs and field settings have been helping students build SW practice competencies using virtual platforms. While remote learning has existed in varied formats for many years, there have been questions about how we can effectively teach SW practice online (Knowles, 2007; Levin et al., 2018). SW practice learning requires *practice opportunities* for students, as these will enable instructors to assess *demonstrated* competencies in addition to their knowledge acquisition of theoretical frameworks and concepts related to SW practice. Through observation of practice (e.g. role-play and simulation), instructors can provide feedback as well as demonstrate and coach practical skills and guide students’ reflections on practice. In a study with SW faculty (*N* = 376), Levin et al. (2018) found that despite rapid advances in technology, faculty concerns about the effectiveness of online teaching versus traditional teaching have not changed over the years, especially related to practice. Faculty reported that online learning is less effective than in-person learning in preparing students for practice. They identified concerns about effectively teaching *use of self* and *therapeutic alliance* virtually. In a study on e-learning with Canadian SW faculty and administrators, Knowles (2007) identified several challenges and grouped them in four categories to serve as a pedagogical framework for policy and implementation for online teaching in SW: (1) pedagogical challenges (e.g. online relationship building, redesigning curricula and teaching methods), (2) professional challenges (e.g. alignment of e-learning with SW education, ethics, professionalism, and e-equity), (3) faculty challenges (e.g. time, workload, compensation, professional development, and technical support), and (4) administrative challenges (e.g. program structure and academic policies). Building on Knowles’ framework, East et al. (2014) recommend developing communities of practice for online learning and emphasize that this is transformative change with a need for ‘institutional support and resources, leadership, faculty engagement, and a pedagogical alignment that is unique to a practice profession’ (p. 31).

Faculty concerns about online learning for practice courses and clinical competencies were documented pre-pandemic. However, there has been recent feedback from students experiencing the effects of the COVID-19 pandemic. The CSWE survey on student perceptions (*N* = 3564) showed that most students (80%) prefer in-person classes and 61 percent felt they learn less through online teaching (CSWE, 2020). This leaves us with questions on how to address these challenges effectively to prepare students for SW practice when online teaching is the only medium available during the pandemic. During this unsettling and traumatic period, our students are receiving less practice training, while our communities need more assistance from social workers. Therefore, we would like to invoke educators to reflect on this dialectic between the limited evidence and needs in the field, and how we can optimize learning in the current context.

We will share two examples of our discoveries as we learned how to effectively teach SW practice competencies during COVID-19. Our first example outlines a voluntary educational enhancement known as *Virtual Practice Fridays*, which was adapted to an online format to provide students with ongoing practice after experiencing disruptions to practica during the pandemic. The second example describes a re-designed course on *cross-cultural SW practice* offered virtually for the first time in spring 2020.

### Virtual Practice Fridays

Practice Fridays is a voluntary simulation-based learning activity launched in 2015 to provide our students with more opportunities to develop SW practice competencies. This teaching innovation includes a small group of students, a simulated client played by an actor, and a faculty member or PhD student facilitator (Kourgiantakis et al., 2020). During the pandemic, we collaborated with our field office and offered Virtual Practice Fridays to two groups of Master of Social Work students with 10 students in each group, for 10 weeks, lasting 3.5 hours per session. The first session focused on the introduction of group members and facilitators, the aim and structure of Virtual Practice Fridays, group norms and policies, practice skills, as well as assessments and case formulations. Sessions 2–9 were assessment, brief intervention, and termination sessions with the simulated clients. Students were in the role of the social worker and they also observed their peers interacting with the simulated client. We situated both scenarios and clients in the current context of the COVID-19 pandemic and the global movement for anti-racism.

Participating students developed procedural competencies as they practiced assessment, interviewing, and alliance-building skills. Students received feedback on skills from peers and group facilitators. Facilitators provided *mini-lecturettes* on topics including case conceptualization and evidence-based practices, and they helped students gain a better understanding of their values, assumptions, and strengths. In between sessions, students completed written client assessments, case notes, process recordings, and other exercises that enhanced critical reflection and knowledge about SW practice. These activities helped students enhance metacompetence, including self-awareness, self-reflection, emotion regulation, and professional judgment. They completed reflection questionnaires requiring them to critically reflect on their own cultural identities, as well as those of their client, and how these intersectional identities impact the helping relationship. As the faculty member overseeing Virtual Practice Fridays, the first author provided weekly supervision to the PhD student facilitators.

### Cross-cultural SW practice

The second author designed a course on cross-cultural SW practice course to increase reflexivity of students when working cross-culturally and to enhance students’ competence in socially just and culturally competent practice (SJCCP). The course included several teaching methods to enhance students’ metacompetence (e.g. reflection discussion), and procedural competence (e.g. role-play). During the pandemic, these teaching approaches had to move to a virtual space, and in order to increase students’ *metacompetence in SJCCP* we implemented three approaches. First, we created an online discussion board to build relationships among classmates, so students feel less guarded to share self-reflections on cultural biases and prejudice, racial privileges, and unmarked Whiteness norms. Second, we used a blended learning approach combining synchronous (i.e. virtual class) and asynchronous (i.e. self-guided practice learning) formats. The final approach was students’ critical reflections on the *digital story telling* of a simulated client, describing their concerns and showing cross-cultural complexities of SW practice. Students developed a cultural formulation of presenting issues and a critical analysis using anti-oppressive approaches. They also engaged in critical self-reflection on cross-cultural similarities and differences with the simulated client, and how this impacts relationship building and treatment.

In order to foster students’ *procedural competencies in SJCCP*, we used three scaffolded approaches. First, students engaged in role-plays during virtual classes using breakout rooms. We also had a virtual session with a simulated client presenting with complex cultural and systemic issues with a similar format to Virtual Practice Fridays. Finally, students filmed a role-play that demonstrated approaches they would use with the client they had learned about through the digital story-telling video.

## Conclusion

This brief note has discussed a disruption to SW education and practice engendered by the pandemic. As illustrated through two examples, we have been trying to adjust and provide expedited alternatives to the changes evoked by the pandemic, while also seeking to identify best practices in teaching SW practice virtually. We summarize three discoveries about teaching SW practice online: (1) online education can be effective if/when faculty and leadership take into account factors such as time, workload, policies, organization, and curriculum; (2) there has not been enough research on *how* to effectively teach SW practice online and some studies have raised concerns about its effectiveness (East et al., 2014; Levin et al., 2018); and (3) blended learning approaches show promising results for teaching SW practice as students can learn about theoretical frameworks online and apply this knowledge in the classroom.

## References

[bibr1-0020872820959706] Ayala J. Drolet J. Fulton A. Hewson J. Letkemann L. Baynton M. Elliott G. Judge-Stasiak A. Blaug C. Tétrault Gérard A. Schweizer E. (2018) ‘Field Education in Crisis: Experiences of Field Education Coordinators in Canada’, Social Work Education 37(3): 281–93.

[bibr2-0020872820959706] Canadian Association for Social Work Education (CASWE) (2020) Statement on the Critical Role of Field Education in Social Work Education. Ottawa: CASWE.

[bibr3-0020872820959706] Council on Social Work Education (CSWE) (2015) Educational Policy and Accreditation Standards. Alexandria, VA: CSWE.

[bibr4-0020872820959706] Council on Social Work Education (CSWE) (2020) Social Work Student Perceptions: Impact of COVID-19 Pandemic on Educational Experience and Goals. Alexandria, VA: CSWE.

[bibr5-0020872820959706] East J.F. LaMendola W. Alter C. (2014) ‘Distance Education and Organizational Environment’, Journal of Social Work Education 50(1): 19–33.

[bibr6-0020872820959706] Knowles A.J. (2007) ‘Pedagogical and Policy Challenges in Implementing E-Learning in Social Work Education’, Journal of Technology in Human Services 25(1–2): 17–44.

[bibr7-0020872820959706] Kourgiantakis T. Sewell K.M. Hu R. Logan J. Bogo M. (2020) ‘Simulation in Social Work Education: A Scoping Review’, Research on Social Work Practice 30(4): 433–50.

[bibr8-0020872820959706] Lai J. Ma S. Wang Y. Cai Z. Hu J. Wei N. Wu J. Du H. Chen T. Li R. Tan H. Kang L. Yao L. Huang M. Wang H. Wang G. Liu Z. Hu S. (2020) ‘Factors Associated with Mental Health Outcomes among Health Care Workers Exposed to Coronavirus Disease 2019’, JAMA Network Open 3(3): e203976. 10.1001/jamanetworkopen.2020.3976 PMC7090843 32202646

[bibr9-0020872820959706] Lee E. Bogo M. Tsang A.K.T. (2020) ‘A Socially Just and Culturally Competent Clinical SW Practice’, in Brandell J.R. (ed.) Theory and Practice in Clinical Social Work, pp. 567–88. San Diego, CA: Cognella.

[bibr10-0020872820959706] Levin S. Fulginiti A. Moore B. (2018) ‘The Perceived Effectiveness of Online Social Work Education: Insights from a National Survey of Social Work Educators’, Social Work Education 37(6): 775–89.

[bibr11-0020872820959706] Serhan Y. McLaughlin T. (2020) ‘The Other Problematic Outbreak: As the Coronavirus Spreads across the Globe, So Too Does Racism’, The Atlantic, 13 March. Available online at: https://www.theatlantic.com/international/archive/2020/03/coronavirus-covid19-xenophobia-racism/607816/ (accessed 10 August 2020)

[bibr12-0020872820959706] World Health Organization (WHO) (2020) Mental Health and Psychosocial Considerations during the COVID-19 Outbreak. Geneva: WHO.

